# Therapeutic potential of synergistic mucociliary clearance for cystic fibrosis airways by β-adrenergic plus cholinergic agonists

**DOI:** 10.1172/JCI201541

**Published:** 2026-04-02

**Authors:** Nam Soo Joo, Susan E. Birket, Johnathan D. Keith, Juan P. Ianowski, Xiaojie Luan, Jacquelyn Spano, Jennifer B. Bollyky, Marissa N. Dobry, Juan R. Sabater, Ryan W. Williams, John F. Engelhardt, Jeffrey J. Wine, Carlos E. Milla

**Affiliations:** 1 The Cystic Fibrosis Research Laboratory and; 2 Center for Excellence in Pulmonary Biology, Stanford University, Stanford, California, USA.; 3 Department of Medicine, Heersink School of Medicine, University of Alabama at Birmingham, Birmingham, Alabama, USA.; 4 Department of Anatomy, Physiology and Pharmacology, College of Medicine, University of Saskatchewan, Saskatoon, Saskatchewan, Canada.; 5 Department of Medicine and; 6 Innovative Medicines Accelerator Program, Stanford University, Stanford, California, USA.; 7 Friedman Advanced Research Institute, Mount Sinai Medical Center, Miami, Florida, USA.; 8 Department of Anatomy and Cell Biology, Carver College of Medicine, University of Iowa, Iowa City, Iowa, USA.; 9 Division of Pulmonary, Allergy and Critical Care Medicine, University of Alabama at Birmingham, Birmingham, Alabama, USA.

**Keywords:** Cell biology, Clinical Research, Pulmonology, Chloride channels, Epithelial transport of ions and water, Pharmacology

## Abstract

Mucociliary clearance (MCC) is an innate defense mechanism that normally keeps airways clean but is dysfunctional in cystic fibrosis (CF) and other muco-obstructive pulmonary diseases. Previously we discovered that activating adenyl cyclase in combination with a cholinergic agonist increased MCC velocity (MCCV) synergistically in ex vivo WT and CF ferret and WT piglets. For what we believe is the first time, we show in vivo synergistic MCC using FDA approved β-adrenergic and cholinergic drugs delivered to the apical surface of WT and CF rats and a CF sheep model. Also, a single dose of the combined drugs is tolerated by humans. As for mechanisms, via ex vivo experiments, we show the combined agonists increased net fluid secretion mainly by stimulating gland secretion and by inhibiting surface absorption, consequently increasing airway surface liquid depth. They also increased net base secretion and increased ciliary beat frequency. Additional ex vivo and in vitro experiments show that the combined agonists had additive effects when combined with highly effective CF transmembrane conductance regulator modulator therapy. The synergistic increase in MCCV induced by this combination of agonists offers therapeutic potential for treating muco-obstructive pulmonary diseases, including CF.

## Abstract 2

Repurposing clinically approved, inexpensive drugs is faster and cheaper than development of new therapeutic entities. Further development of synergy agonists provides an attractive additional approach toward alleviating CF and possibly other muco-obstructive airway disorders.

## Graphical abstract

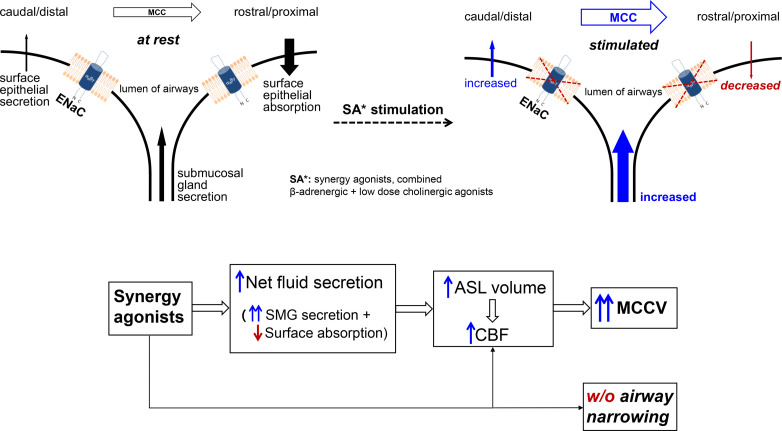

## Introduction

Mucociliary clearance (MCC) plays a pivotal role in host innate defense of the airways. Impaired mucus clearance contributes to cystic fibrosis (CF) and other obstructive airway diseases (1–3). In CF, defective MCC results from deficient CF transmembrane conductance regulator (CFTR) function that can now be treated with highly effective CFTR modulator therapies (HEMTs) (4–6). These provide significant clinical benefits to most people with CF (pwCF), but ~10%–20% of pwCF show no or insufficient benefits for a variety of reasons (7, 8). For them, other means are needed to improve MCC. Various approaches have had some success in improving MCC — e.g., dornase-α (9) and hypertonic saline (10) — but more efficacious treatments are necessary.

In prior work with ex vivo tracheae from WT and CF ferrets and newborn piglets, we discovered that very large increases in MCC velocity (MCCV) were produced by combined treatment with the adenylyl cyclase activator 10 μM forskolin and the cholinergic agonist 0.3 μM carbachol (11). Each of these alone increases MCCV somewhat, but together they exhibit powerful synergism that increases MCCV to near-maximum values, without causing bronchoconstriction (12). Of note, synergy was observed in transgenic CF ferret tracheal preparations, despite no increase in MCCV with forskolin alone (12).

To enable eventual therapeutic application of this powerful synergistic increase in MCCV, multiple questions that were not addressed in earlier work needed to be answered. Our prior experiments were done ex vivo through basolateral administration and using pharmacologic agonists that are not approved for human use. These were applied sequentially to the basolateral bath and were then present throughout the measurement period. We did not test for potential tolerability, nor did we determine interactions with HEMT.

In the present study we used the clinically approved β-adrenergic agonist formoterol instead of forskolin and methacholine instead of carbachol. Airway apical surface delivery of the agonists was effective in 2 in vivo CF animal models (sheep and rats). The increased MCCV in the sheep model was prolonged for approximately 24 hours. We found that simultaneous application of the 2 agonists speeded MCCV as effectively as sequential application and without airway constriction. When combined with HEMT, the agonists caused further increases in MCCV. We suggest potential mechanistic insights into how the combined agonists induced synergistic MCC. Finally, in a pilot study with healthy volunteers and pwCF, we corroborated the tolerability of a single nebulized dose of the combination at different doses. These results resolve some of the uncertainties regarding the effects in the airway and tolerability of these combined agonists and speak of their great potential to alleviate mucus obstruction.

## Results

### Formoterol and methacholine applied sequentially or simultaneously increase MCCV without causing airway narrowing.

**Figure 1:**
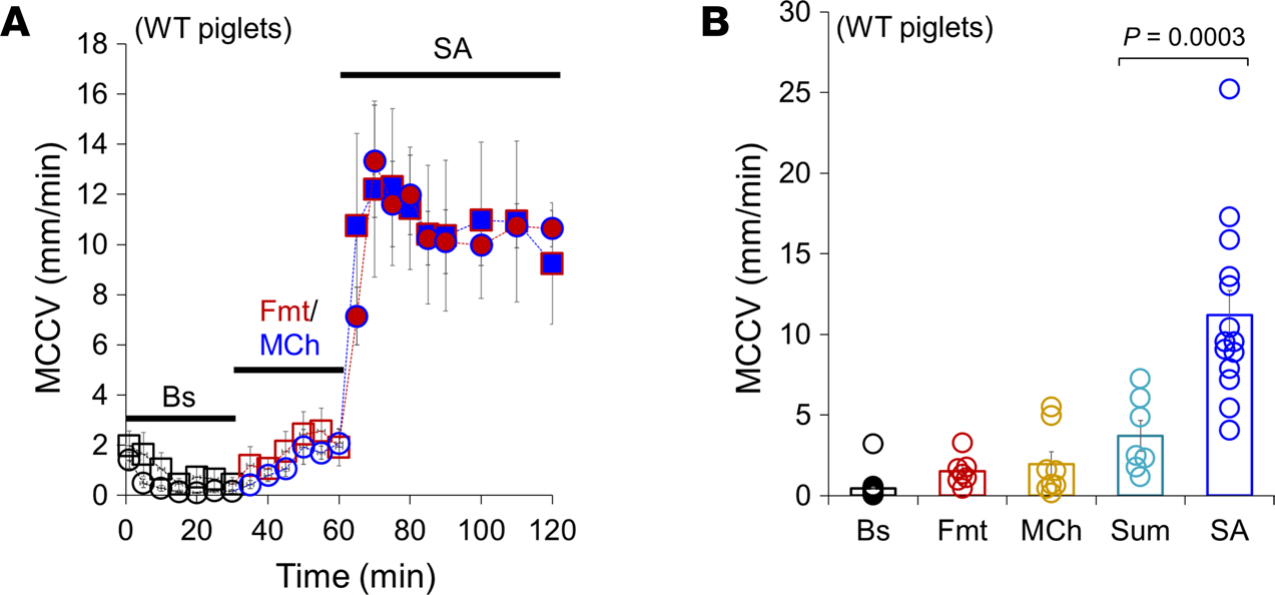
MCCV measured in ex vivo animal tracheae exposed to formoterol, methacholine, or the combination in either order. (**A**) Time courses of ex vivo MCCV showing synergistic MCC by the combined (synergy agonists, SA) 10 μM formoterol and 0.3 μM methacholine when formoterol (Fmt, red circles) or methacholine (MCh, blue squares) was added sequentially after baseline (Bs) in newborn piglet tracheae (*n* = 7–15 newborn piglets). (**B**) Summary of piglet MCCV data by SA: Fmt, 10 μM formoterol alone; MCh, 0.3 μM methacholine alone; Sum, arithmetic sum of MCCV by Fmt alone plus MCh alone. The mean values were from periods as follows: Bs, T10-T30; Fmt or MCh, T10-T30 (or T40-T60, time course); and SA (Fmt + MCh), T10-T60 (or T70-T120, time course). A 1-way ANOVA with Bonferroni’s post hoc test was used.

For direct comparison with prior ex vivo experiments using forskolin and carbachol (11, 12), we sequentially applied formoterol, low-dose methacholine, or the combination to the serosal bath of 3- to 5-day-old WT piglet tracheae. These compounds are both approved for inhalation, though methacholine is at present approved only for single administration as a test for airway reactivity. Formoterol (10 μM) or methacholine (0.3 μM) alone each increased MCCV, while in combination they increased MCC to a velocity *far greater* than the simple addition of the responses to the individual agonists (Figure 1A). Because of this synergistic effect, we refer to the combination as “synergy agonists” (SA). Figure 1B shows mean MCCV values for each animal and group for each condition. Numerical MCCV values (mm/min) of baseline and single and combined drugs are provided in Supporting Data Values; supplemental material available online with this article; https://doi.org/10.1172/JCI201541DS1 The MCCV induced by SA was significantly larger than that of the arithmetic sum of responses to individual agonists (*P* = 0.0003, *n* = 7–14).

**Figure 2:**
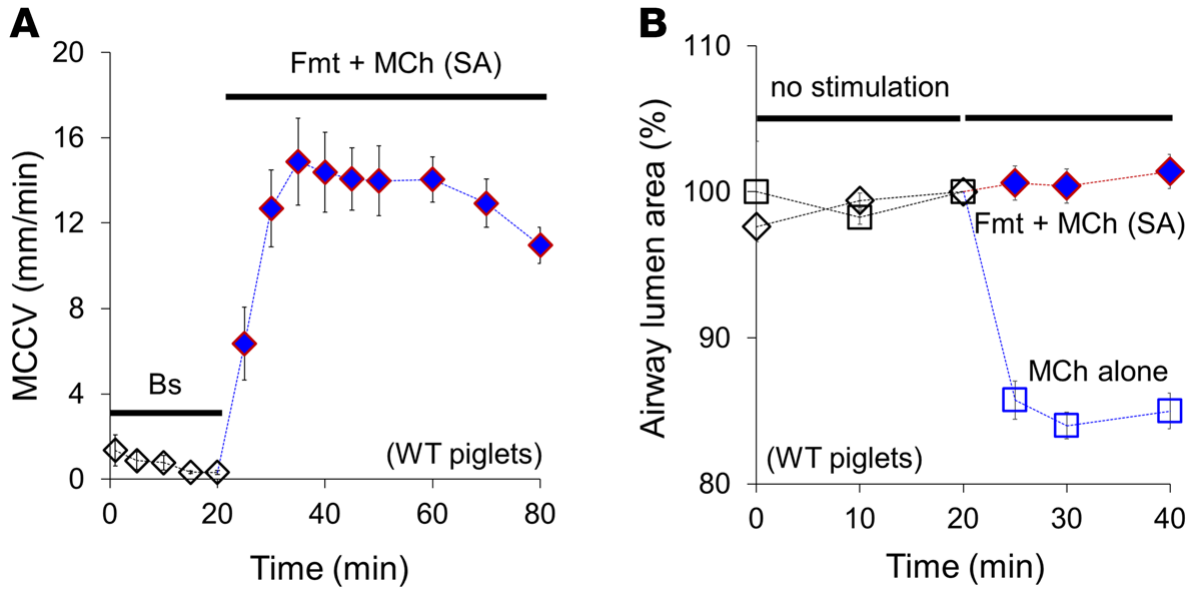
MCCV and lumen area measured in ex vivo animal tracheae before and after *simultaneous* formoterol and methacholine. (**A**) A time course of ex vivo MCCV showing the simultaneous SA (10 μM formoterol, Fmt, + 0.3 μM methacholine, MCh) addition after baseline (Bs) period without preexisting agonist produces a comparable MCCV (*n* = 6 newborn piglets). (**B**) Simultaneous SA (Fmt + MCh) after baseline (black diamonds) did not cause airway narrowing, while methacholine alone induced airway narrowing (blue squares) in ex vivo piglet tracheal preparations (*n* = 4–5 newborn piglets).

Sequential addition of agonists is useful for quantifying the extent of synergy. However, simultaneous administration, which is preferable for eventual clinical development, might produce different effects. But as shown in Figure 2A, simultaneous administration of both agonists combined as SA was just as efficacious in increasing MCCV (in mm/min): from 0.5 ± 0.2 at baseline to 13.5 ± 0.5, *n* = 6 piglets (compare Figure 1A and Figure 2A). As with sequential addition, simultaneous addition did not induce airway narrowing (Figure 2B and Supplemental Figure 1), whereas MCh alone reduced the luminal area to 84.9% ± 0.4% of starting value.

### Increased net base secretion, decreased fluid absorption, and increased airway surface liquid volume following SA.

**Figure 3:**
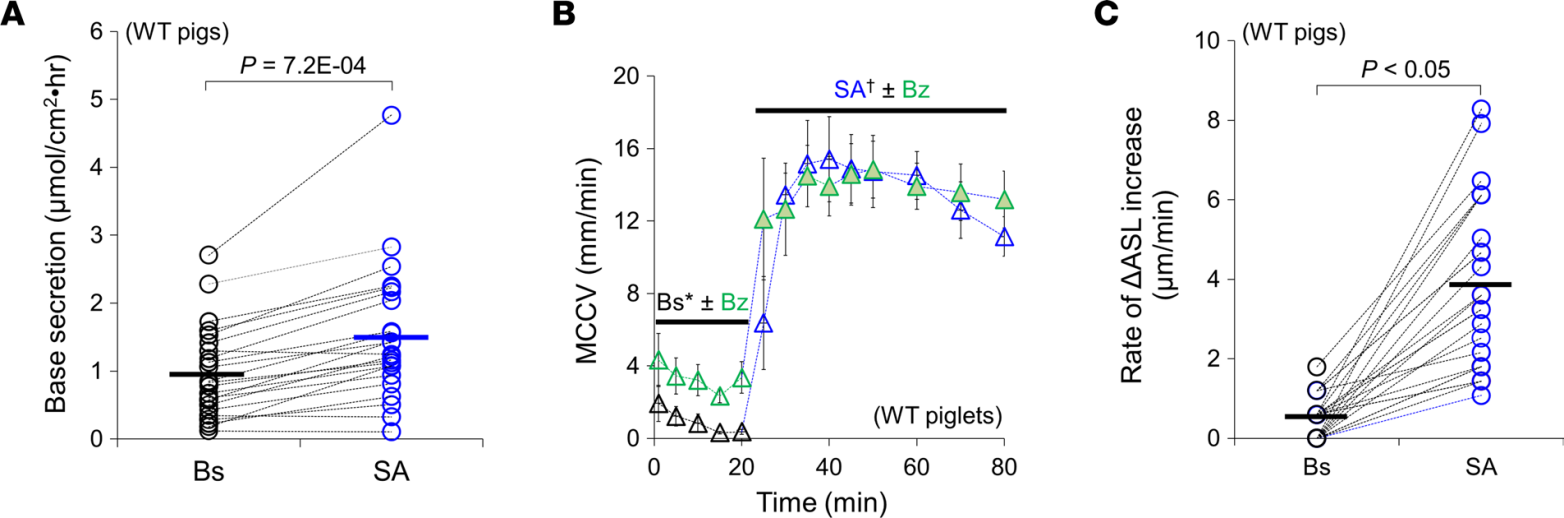
Formoterol and methacholine increase net base secretion, inhibit fluid absorption, and increase ASL depth. (**A**) Base secretion was assessed by pH-stat method. SA significantly increases net base production compared with baseline in freshly isolated WT pig tracheal mucosa (*n* = 23 tracheal tissue preparations from 16 pigs). A 2-tailed paired *t* test was used. (**B**) Time courses of ex vivo MCCV showing ENaC inhibition by 10 μM benzamil (Bz) significantly increased baseline MCCV with no additional effect on MCCV induced by SA regardless of benzamil treatment in ex vivo tracheal MCCV measurements in piglets (*P = 0.02, *n* = 4 each condition). SA^†^,(*P* = 0.97 with a 2-tailed unpaired *t* test) or simply not significant. (**C**) SA (10 μM formoterol + 0.3 μM methacholine) significantly increased the rates of ASL depth changes from baseline (Bs) in ex vivo pig tracheae, assessed by phase contrast imaging using synchrotron x-rays (*P* = 0.05 with a 2-tailed paired *t* test, 24 beads from *n* = 4 pigs). The ASL depth change (in μm): 102.4 ± 8.7 during T20 baseline period and 251.4 ± 19.6 during T30 SA treatment period.

We hypothesize that the combined agonists accelerate MCCV via an increase in airway surface liquid (ASL) volume, as well as increasing ciliary beat frequency (CBF) and increasing pH by net base (most likely, bicarbonate — see Discussion) secretion. Mucus pH is relevant because viscoelasticity increases and MCC slows when mucus becomes more acidic (13, 14). To assess whether SA increases HCO_3_^–^ secretion in airways, freshly isolated pig tracheal mucosa were mounted in Ussing chambers equipped for pH-stat measurement (15). Addition of SA significantly increased net base secretion (in μmol/cm^2^•hr) from 1.0 ± 0.1 at baseline to 1.5 ± 0.2 (*P* = 7.2 × 10^–4^, 23 tissue preparations from 16 pigs) (Figure 3A).

In a previous study, we showed that forskolin and carbachol synergistically increased submucosal gland fluid secretion and decreased epithelial sodium channel–mediated (ENaC-mediated) short-circuit current (I_sc_) (12), which should increase ASL volume. We expected that addition of SA would replicate these effects. However, in prior work, we did not measure ASL volume directly. To assess the effect of SA on fluid absorption and the role of decreased absorption on MCCV, we measured baseline and SA-induced MCCV in the presence or absence of benzamil (10 μM), an ENaC blocker. In ex vivo piglet tracheae, as shown in Figure 3B, benzamil treatment significantly increased baseline MCCV (in mm/min): 0.5 ± 0.2 at baseline + vehicle (0.1% DMSO) versus 3.0 ± 0.7 baseline + benzamil (*P* = 0.02, 4 piglets each condition) (Supplemental Methods Protocol 1). When SA was added to the benzamil-treated tissue, it caused the expected large increase in MCCV. However, that increase was not significantly larger than the increase observed in the absence of benzamil: MCCV in mm/min: 14.0 ± 1.6 with SA + vehicle versus 13.9 ± 1.6 with SA (*P* = 0.97, 4 piglets each condition).

To measure ASL depth, we applied synchrotron-based phase contrast imaging (see Methods) to ex vivo pig tracheae either unstimulated or stimulated with SA. As shown in Figure 3C, ASL depth increased at a significantly faster rate following SA (*P* < 0.05, 24 beads from *n* = 4 pigs) (see Discussion).

### In vivo studies of SA in 2 CF animal models.

Up to this point, all experiments were conducted with ex vivo tracheal preparations. These lack the normal baseline activity of the autonomic regulation of the airways (16) and do not assess possible effects on distal airways. Drugs were typically applied basolaterally and were continuously present. Finally, the mucosal environment of breathing animals differs from ex vivo conditions. For these reasons, in vivo studies are essential to determine if the results seen ex vivo can be replicated in vivo. We used 2 animal models of CF, sheep with airways exposed to an epithelial CFTR blocker plus recombinant human neutrophil elastase (*h*NE) to induce inflammation (17, 18) and transgenic CF rats (19, 20). For both models, SA were applied in vivo luminally to the apical surface as single doses.

### Sheep model of inflamed CFTR-deficient airways.

**Figure 4:**
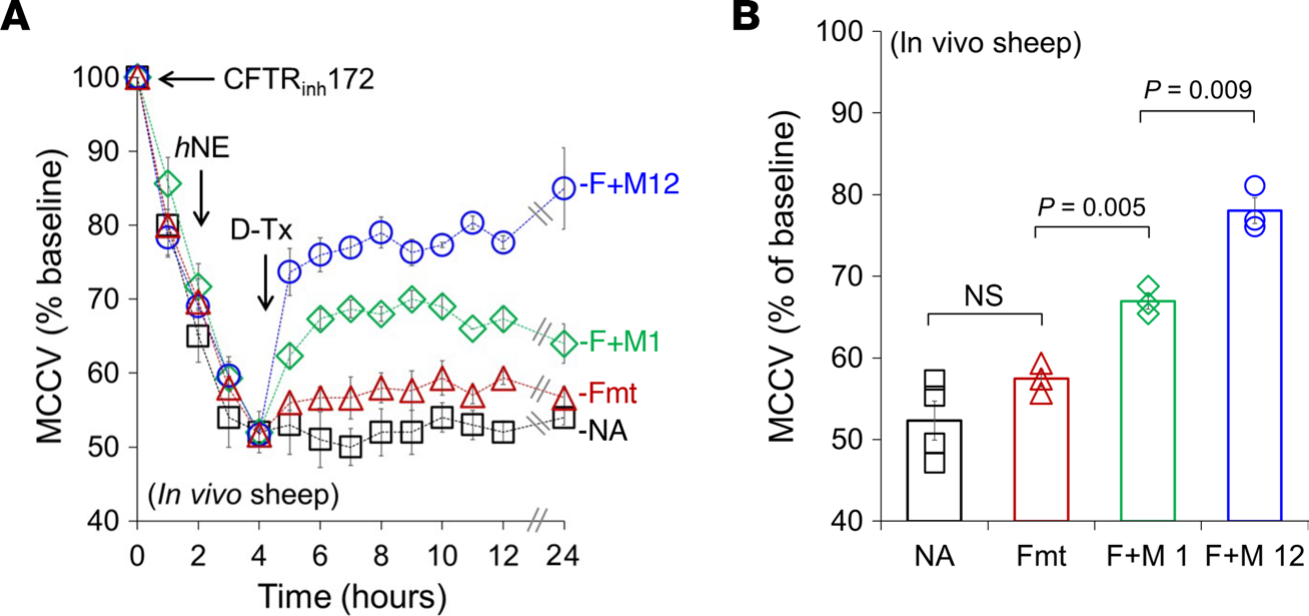
Prolonged increases of MCCV in an in vivo sheep model of inflamed CF airways following nebulization of formoterol and methacholine. (**A**) Time courses of in vivo MCCV showing dose-dependent increases of tracheal MCCV by SA in a sheep model of inflamed CF airways. Symbols and abbreviations: black squares, no agonist (NA); red triangles, 20 μg formoterol (Fmt or F) alone; green diamonds and blue circles, 20 μg Fmt + either 1 or 12 μg methacholine (M); *h*NE, recombinant human neutrophil elastase; D-Tx, drug treatment. (**B**) Summary of in vivo sheep data: no agonist, 20 μg formoterol alone, SA (formoterol + 1 μg methacholine/M 1 or formoterol + 12 μg methacholine/M 12) (*n* = 3–4 sheep). Note that while formoterol alone was not different in MCCV from vehicle-treated sheep (NA), F + M12 and F + M1 produced significantly larger MCCV than F + M1 and Fmt alone, respectively. A 1-way ANOVA with Bonferroni’s post hoc test was used.

To approximate inflamed CF airways, WT sheep were nebulized with 10 mg CFTR_inh_172 followed 2 hours later by 2,380 milliunits of *h*NE. This treatment suppressed MCCV to approximately 50% of baseline at 4 hours from the start of the experiment, and this effect was sustained for at least ~20 hours (in %) (52.3 ± 2.4, 4 sheep). At *t*4 hours, when MCCV was maximally slowed, nebulization of 20 μg formoterol did not significantly increase MCCV. However, SA of 20 μg formoterol in combination with either 1 or 12 μg methacholine produced marked, sustained increases in MCCV (Figure 4A). As shown in the summary data (Figure 4B), a dose-dependent effect was noted (in % from baseline): 20 μg formoterol alone did not increase tracheal MCCV (*P* = 0.12 to baseline, *n* = 3 sheep); however, both SA of 20 μg formoterol + 1 μg methacholine (*P* = 0.005 vs. Fmt alone, *n* = 3 sheep) and SA of 20 μg formoterol + 12 μg methacholine significantly increased MCCV (*P* = 0.009 vs. 20 Fmt + 1 MCh, *n* = 3 sheep). Surprisingly, a single nebulization of SA had a sustained effect with an improvement in MCC for at least 20 hours. This contrasts with ex vivo experiments with piglets, where in the constant presence of SA, MCCV declined by approximately 50% from peak velocity in only 2 hours (Supplemental Figure 3) (see Discussion).

### CFTR-null rats.

**Figure 5:**
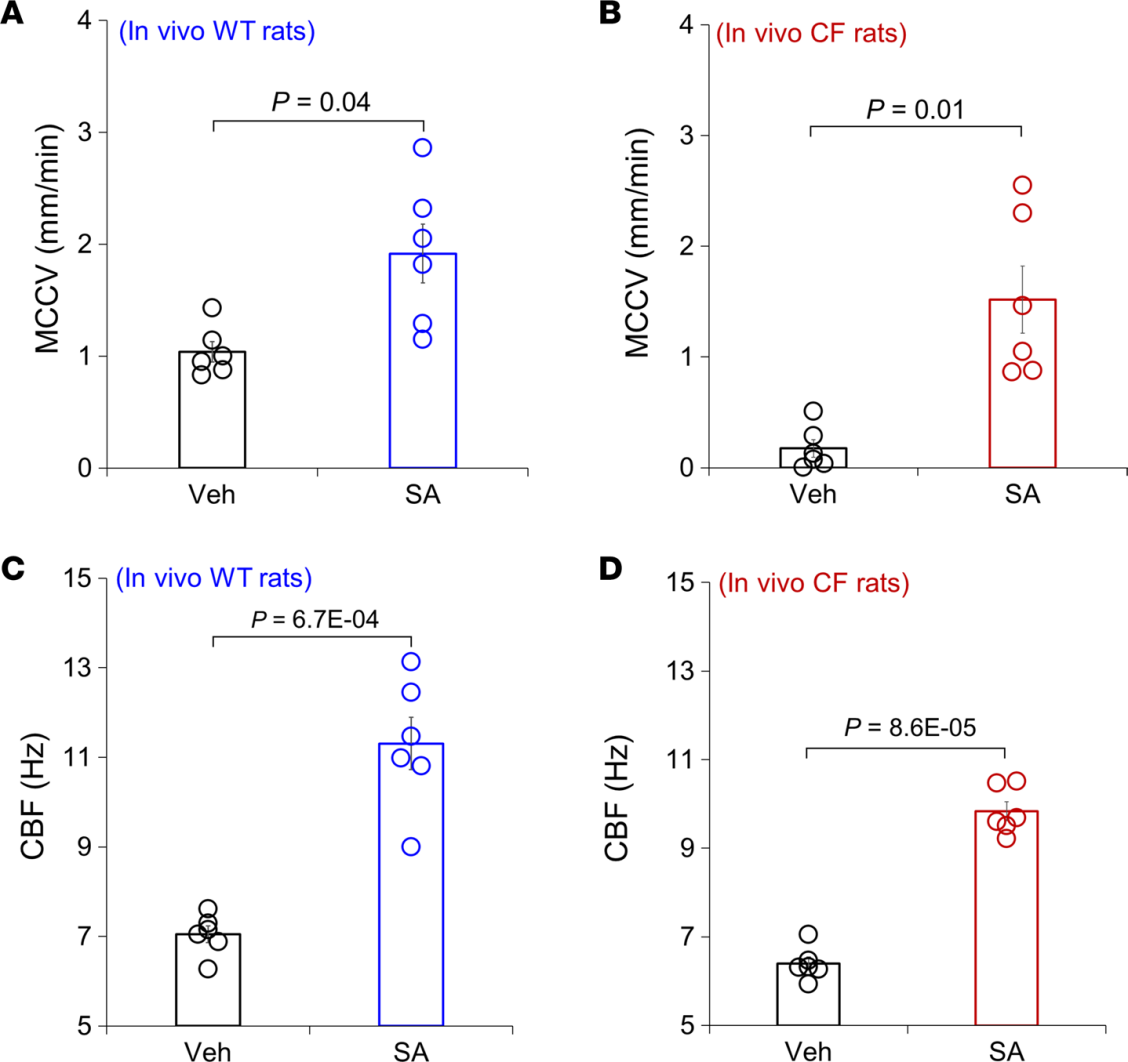
MCCV and CBF measured in ex vivo tracheae of WT and CFTR-*null* rats after in vivo intratracheal instillation of vehicle or formoterol and methacholine. SA (15 μg formoterol + 0.5 μg methacholine) significantly increase MCCV, measured by μOCT, in both WT (**A**) and *CFTR-null* rats (**B**) compared with their vehicle-treated (saline) baseline (Veh) groups (*n* = 6 rats each condition). Note that vehicle-treated MCCV in CF rat tracheae was significantly reduced compared with that of WT rats. Also notice that SA increased tracheal MCCV in CFTR-*null* rats to ~80% of that of WT by SA and more than that of vehicle-treated WT. (**C** and **D**) SA significantly increased CBF, measured simultaneously with tracheal MCCV by μOCT, compared with that of the vehicle-treated group. All comparisons were done with a 2-tailed unpaired *t* test.

The sheep model has advantages but is not a product of absent CFTR gene function, so we also examined the effects of SA administration on tracheal MCCV and CBF in WT rats and in transgenic CFTR-knockout (CFTR-*null*) rats. We used a minimally invasive method to instill SA of 15 μg formoterol + 0.5 μg methacholine into tracheae of WT and CFTR-*null* rats. After 30 minutes of SA administration, the animals were euthanized, and tracheal MCCV and CBF were measured ex vivo via micro-optical computed tomography (μOCT) imaging. As shown in Figure 5, A and B, MCCV was significantly increased by SA in both WT and CF, when compared with the vehicle-treated (saline) groups: WT (SA, *P* = 0.04, *n* = 6 rats) and CF (SA, *P* = 0.01, *n* = 6 rats). In explanted tracheae from vehicle-treated CF rats, MCCV was only 16.3% of WT (*P* = 2.7 × 10^–5^, *n* = 6 each condition), while the fold-increase in SA-induced MCCV was much larger in the CF animals, so SA-induced MCCV did not differ significantly between WT and CF (*P* = 0.34, *n* = 6 each condition). CBF was also significantly increased by SA treatment in both WT (SA, *P* = 6.7 × 10^–4^, *n* = 6 rats) and CF (SA, *P* = 8.6 × 10^–5^, *n* = 6 rats) (Figure 5, C and D). In vehicle-treated rats, CBF of CF was slightly, but significantly, reduced compared with that of WT (*P* = 0.02, *n* = 6 rats each condition). Quantitative data for MCCV and CBF in response to vehicle and SA treatments are provided in Supporting Data Values.

### SA increase MCCV in ivacaftor-treated CF ferrets.

**Figure 6:**
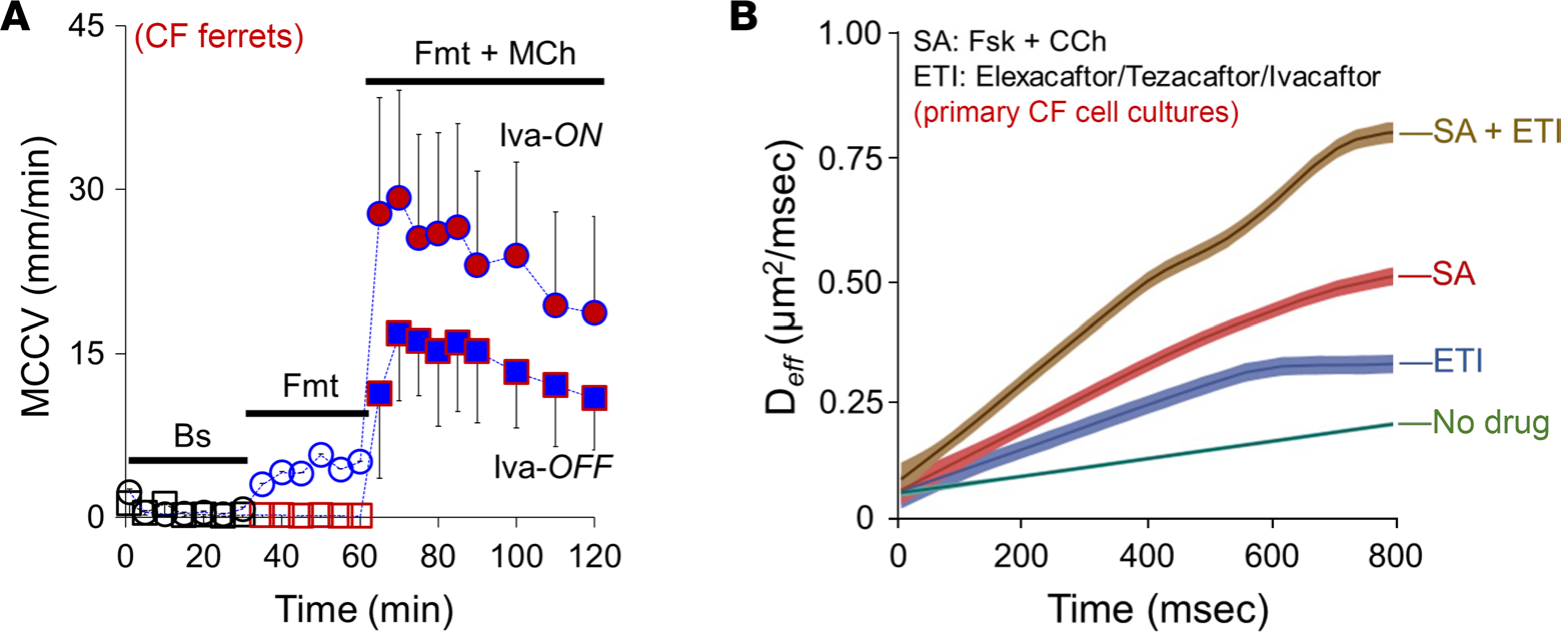
Formoterol and methacholine provide additional benefits when combined with HEMT. (**A**) Time courses of ex vivo MCCV showing synergistic MCC by SA (10 μM formoterol + 0.3 μM methacholine) in CFTR*^G551D^* CF ferret tracheae ± ivacaftor (*n* = 3–4 CF ferrets). Note that when CF ferrets had been off ivacaftor (>30 days, *Iva-OFF* at the bottom MCCV trace), formoterol did not have an effect on MCCV (red squares), while ferrets continuously administered ivacaftor until their euthanasia showed increased MCCV by 10 μM formoterol (*Iva-ON* at the top MCCV trace). Also notice that SA increased MCCV in both groups of ferrets, while *Iva-ON* ferrets produced larger MCCV increases compared with those of *Iva-OFF* ferrets. (**B**) Measured in vitro particle D*_eff_* (in μm^2^/msec) from primary human nasal CF (CFTR*^delF508^* homozygous) cells with and without HEMT, ETI (3 μM elexa/3 μM teza/10 μM ivacaftor). Increasing D*_eff_* represents actively transported particles by the mucus flow, with steeper tracings reflecting increasingly facilitated transport by a more fluid system. Note that SA (10 μM forskolin + 0.3 μM carbachol) further increased D*_eff_* when combined with ETI (*n* = 3).

To determine how SA and HEMT might interact, we compared MCCV between CFTR*^G551D^* ferrets (21) continuously treated or not with ivacaftor for at least 30 days prior to euthanasia. Tracheae were removed and treated ex vivo with SA. As expected, tracheae from untreated ferrets had no CFTR function and did not increase MCCV in response to formoterol, while tracheae from ivacaftor-treated ferrets did (in mm/min): 0.4 ± 0.3 untreated vs. 4.4 ± 1.0 treated with ivacaftor (Figure 6A). In contrast, SA produced large, synergistic increases in tracheal MCCV in both ivacaftor-treated and untreated ferrets (in mm/min): 14.5 ± 9.1 with untreated vs. 24.0 ± 5.3 treated with ivacaftor. MCCV by SA in ivacaftor-treated ferrets was ~1.7-fold greater than in untreated ones, reflecting the contribution of CFTR and indicating an additional benefit of SA on HEMT-treated CF animals.

### SA added to HEMT further increases mucus transport in vitro in cultured CF human nasal cells.

As a further test of additivity between SA and HEMT, we used high-speed digital microscopy to estimate the effective diffusivity (D*_eff_*; in μm^2^/msec) of fluorescent polystyrene spheres added to the ASL layer of human CF (F508del homozygote) primary nasal cell cultures grown at air-liquid interface (ALI) conditions ± HEMT triple therapy (ETI; 3 μM elexacaftor + 3 μM tezacaftor + 10 μM ivacaftor). As shown in Figure 6B, SA (in this experiment 10 μM forskolin + 0.3 μM carbachol) further facilitated particle transport as evidenced by the increased D*_eff_*.

### Humans tolerate SA.

**Figure 7:**
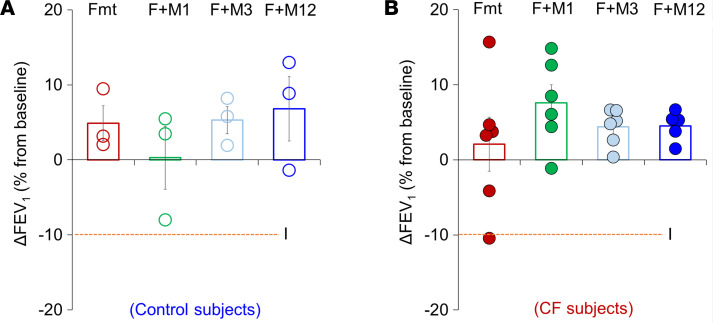
Single-dose formoterol and methacholine does not reduce FEV_1_ at any tested level of methacholine in healthy control or CF individuals. Single-dose trial assessed for tolerability by ΔFEV_1_ after administration by nebulization of 20 μg formoterol alone or SA (20 μg formoterol [Fmt or F] + 1, 3, or 12 μg methacholine [M]) in healthy controls (**A**, *n* = 12) or in people with CF (**B**, *n* = 24). No individual receiving SA met criteria for intolerance (I, orange dotted line), but 1 CF individual did to Fmt alone.

A valid concern with the drugs being evaluated in our studies is the possibility that the methacholine included in the SA combination might cause bronchospasm in spite of its low dose and the demonstrated protective effect of the β-adrenergic agonist in our experimental work. As a preliminary assessment of this concern, we explored the safety of a single dose of nebulized SA in healthy volunteers (*n* = 12) and in pwCF (*n* = 24, all on HEMT). Escalating doses of methacholine (0, 1, 3, 12 μg) were administered in combination with a fixed dose of 20 μg formoterol. Changes in forced expiratory volume in 1 second from baseline (ΔFEV_1_) were measured to evaluate for intolerance (defined as a drop larger than 10%). We did not observe intolerance or other adverse effects in any of the individuals assessed (Figure 7, A and B).

## Discussion

In the present study we extended and strengthened our earlier findings. First, we found that simultaneous administration of SA is as effective as sequential addition: no airway narrowing was seen with simultaneous addition, which will be used in clinical trials. Second, in all our previous and current ex vivo MCCV assays, SA were bath-applied basolaterally, but here, for what we believe is the first time, we demonstrated that nebulization of SA to the apical surface of the in vivo airway is just as effective. Third, to our surprise, we found that a single dose of SA to an in vivo sheep model of CF produced a strong and sustained improvement in MCC. Fourth, we present evidence that SA increased net base secretion, ASL volume, and CBF while inhibiting ENaC-driven fluid absorption via ex vivo experiments. We hypothesize that these all contribute to increased MCCV. Fifth, both in vitro and ex vivo experiment results suggest that SA conferred additional benefits when combined with HEMT. Sixth, in healthy volunteers and pwCF on HEMT, a single dose of SA was well tolerated. Further, airway reactivity is common in CF, and in fact, 9 (37%) of the pwCF dosed had a history of an asthmatic component of their disease, reflecting reactive airways. The observation that this subset of patients did not show intolerance is reassuring as to the safety of our approach and will have to be verified through larger studies.

The role of cholinergic systems in airway clearance is controversial. The anticholinergic agent atropine increased MCCV in anesthetized dogs (22). As shown in Supplemental Figure 2, we found that atropine failed to stimulate piglet tracheal MCCV by itself and completely abolished the response to 10 μM methacholine, findings that are consistent with previous studies using atropine in humans and other animals (23–25). Another anticholinergic agent, tiotropium bromide, did not improve lung function in pwCF (26). By contrast, our earlier (11, 12) and current data show that a low dose of a cholinergic drug increases MCCV by itself and with formoterol in ex vivo animal studies.

Interestingly, MCCV measurements in vivo seem to show larger responses to SA than ex vivo measures. For example, MCCV after in vivo SA in the CF rat was approximately 80% WT versus 50%–60% WT with ex vivo SA in CF ferret (12). Also, the large and sustained increases in MCCV in the sheep contrast with ex vivo piglet data, where MCCV declined from its peak by approximately 50% in the next 2 hours (Supplemental Figure 3). Interpretation of the apparent discrepancy between these 2 experiments is complicated by numerous variables, including species, innervation, oxygenation, and natural breathing between in vivo and ex vivo experiments. However, a couple of plausible explanations can be proposed: the ex vivo piglet MCCV experimental conditions might induce adrenergic and cholinergic receptor desensitization (27) or tolerance due to the constant presence of agonists for the extended period; and/or the route of administration (apical by luminal nebulization vs. systemic as implied by the basolateral bath) may determine more sustained effects of the drug activity from its deposition in the mucosal surface.

In the sheep CF model, a surprisingly sustained response in tracheal MCCV was seen after a single nebulization of formoterol and methacholine. Tracheal MCCV in vivo may also reflect whole lung clearance as mucus moves from distal to proximal airways. Consistent with this interpretation, the single SA treatment improved direct measures of whole lung clearance in the sheep model (Supplemental Figure 4A), and in healthy human volunteers, SA improved forced expiratory flow between 25% and 75% of forced vital capacity, used as a measure of small airway function (28) (Supplemental Figure 4B).

Prior work showed that SA inhibited ENaC-dependent absorption in Ussing chamber experiments (12). We previously demonstrated ENaC inhibition increased the agonist-induced (both forskolin and carbachol) MCCV in ferret tracheae (11). In the present study, we provided evidence that strengthens our earlier findings and further explored to find a link between SA inhibition of ENaC and increased MCCV in piglet tracheae. Baseline MCCV was increased ~6-fold by ENaC inhibition, consistent with the previous interpretation that decreased absorption increases ASL volume and MCCV (11, 12). SA-induced MCCV was approximately equal to that of benzamil-treated tissues and was not further increased by ENaC inhibition. We interpret these results as indicating that synergistically increased MCCV includes inhibition of ENaC, explaining why no further increase is observed in the presence of benzamil. We do not, however, rule out the possibility that the MCCV reaches a physical maximum after each treatment.

The direct measurements of ASL depth using synchrotron-based phase contrast imaging is a well-established methodology (29, 30). Our measurements in ex vivo tracheae, however, are more complicated than in vitro ASL measurements because here the mucus is being transported during the experiments, so its depth depends on the rates of production and flow, like measures of river depth. For depths to increase, the net rate of fluid production (equal to secretion minus absorption) must exceed the increase in MCCV produced by the agonists. This complicates the accurate assessment of the changes of ASL depths by SA. Nevertheless, an increase in depth was observed.

Mucus pH and net base secretion influence the viscosity of airway mucus (13, 14). Under our pH-stat experimental conditions described in Methods (voltage-clamped/short-circuited and basolateral to apical bicarbonate gradient where apical solution was HCO_3_^–^-free), the increased net base secretion stimulated by SA would most likely be bicarbonate secretion. However, other possibilities include potential inhibition of H^+^ secretion via H^+^/K^+^-ATPase/ATP12A (31), which we have not yet examined. It was suggested that acidic pH increases mucus viscoelasticity and slows MCC, while a higher pH tends to decrease mucus viscosity (13, 14, 32). In the present study, we found that SA increased net base (bicarbonate) secretion rates and MCCV in ex vivo and in vivo conditions, but we did not concomitantly measure mucus properties. Increases in D*_eff_* observed following SA in primary CF nasal cell cultures could also partially reflect a more favorable fluidity of the apical surface liquid consistent with higher pH effect and improved mucus viscoelasticity. Due to limited availability of fresh CF animal tissues, our studies of SA effects on net base secretion, ASL depth, and MCCV ± ENaC inhibition were carried out only with WT animals.

Both cAMP- and Ca^2+^-mobilizing agents alter CBF (33). CBF of CF airways has been shown for the most part to be normal (34), unlike in primary ciliary dyskinesia, which arises from an innate cilia malfunction caused by mutations in genes coding functional components of motile cilia (35). However, more recent studies indicate that CBF was reduced in vivo in pwCF (36) and in vitro human primary CF cell cultures compared with healthy controls (37, 38). Decreased CBF found in CF airways is not primary but secondary to altered mucus properties and infection/inflammation (38). Consistent with these findings, CBF data in the present study showed that CFTR-*null* rats have slightly, but significantly, lower CBF than that of WT ones. It is of interest to find that SA restored CBF above baseline CBF of WT.

The combined data from the responses to a single SA dose seen in pwCF receiving HEMT, ex vivo CFTR*^G551D^* ferrets + ivacaftor, and in vitro CF cells + HEMT indicate that SA provides additional improvements in MCC when combined with HEMT, suggesting SA may benefit most pwCF, regardless of their CFTR mutations.

In conclusion, adding a cholinergic agonist to a β-adrenergic one seems counterintuitive for treatment of CF, because cholinergic agonists elevate airway smooth muscle intracellular [Ca^2+^]_i_ to cause bronchoconstriction (12) and because fluid secretion mediated by elevated [cAMP]_i_ is lacking in CF airways (39–46). Possibly for these reasons, the therapeutic possibilities of these combined agonists have escaped notice. The precise cellular/molecular mechanisms responsible for the significant increases in MCCV without bronchoconstriction produced by the combined agonists remain to be established. We have shown that SA stimulates glandular fluid secretion, stimulates net base secretion, inhibits surface epithelial fluid absorption, and increases ASL depth and CBF. In contrast with the mainly agonistic effects of SA (β-adrenergic + cholinergic agents) on epithelial cells (12, 33, 41, 47, 48), it antagonizes smooth muscle contraction (49). We anticipate that the resulting mucus is more abundant and more transportable, hence the increase in MCCV. Measurements of mucus properties ± SA are required to further test the hypothesis.

Repurposing clinically approved, inexpensive drugs is faster and cheaper than development of new therapeutic entities. Further development of SA provides an attractive additional approach toward alleviating CF and possibly other muco-obstructive airway disorders.

## Methods

### Sex as a biological variable.

Our study examined both male and female human participants and animals. As sex-specific differences were not observed, data from both sexes were combined for analysis. Consequently, we did not consider sex as a biological variable.

### Airway tissue procurement.

Newborn piglet tracheae (2–5 days old) were freshly dissected at the Swine Teaching and Research Center of University of California, Davis, and transported to the laboratory in cold PhysioSol solution (Hospira). They were used within 24 hours after harvesting. Postmortem (<1 hour) tracheae from young adult male and female *Yorkshire* pigs or mini-Yucatan pigs were from animal facilities at Stanford University.

We used transgenic CF (CFTR*^G551D^*) ferrets that were genetically engineered at the University of Iowa using CRISPR/Cas9 gene editing to express additional mutant CFTR*^G551D^* in the apical membrane available to carry the ivacaftor-responsive human CFTR mutation Gly551Asp (21). The CF ferret tracheae were shipped in DMEM cell culture medium via overnight delivery. We transferred the tissues to ice-cold Krebs-Ringer bicarbonate (KRB) buffer gassed with 95% O_2_ and 5% CO_2_ until use. The KRB buffer contained (in mM) 115 NaCl, 25 NaHCO_3_, 2.4 K_2_HPO_4_, 0.4 KH_2_PO_4_, 1.2 MgCl_2_, 1.2 CaCl_2_, 10 glucose, and 1.0 μM indomethacin, adjusted to pH 7.2 and ~290 mOsm at room temperature by a vapor pressure osmometer, and an aliquot of an added drug was diluted to 1:1,000 to KRB.

### Human tolerability study.

Following a 3×3 dosing cohort design, 12 healthy volunteers older than 18 years of age (Supplemental Table 1) and free from any history of respiratory disease were enrolled sequentially in cohorts of 3 to evaluate the safety and tolerability of a combination of 20 μg formoterol and 1 of 4 ascending doses of methacholine: 0, 1, 3, or 12 μg. The study drug was administered by nebulization with an eflow mesh nebulizer (PARI). We measured acute effects on vital signs, symptoms, and pulmonary function. This study was followed by a study in pwCF with a demonstrated diagnosis of CF, older than age 18 years, in stable condition, and free from acute exacerbation for at least a month prior to enrollment. The study was similar in all aspects to the healthy volunteer study, with the only difference being that dosing cohorts had 6 pwCF (*n* = 24 total).

### Human CF primary nasal epithelial cell cultures at ALI.

Human nasal epithelial cells (HNECs) were obtained from F508del homozygote (CFTR^Δ*F508/Δ*F508^) pwCF and cultured at ALI as per previously published protocol (50). Once cultures were fully ciliated and active mucociliary activity was visualized, typically after 3 weeks, we proceeded to experimental measurements. To assess if there are additional benefits by SA on CF cells pretreated with highly effective CFTR modulators, CF cells were treated with ETI (elexacaftor 3 μM + tezacaftor 3 μM for 48 hours prior to and supplemented by the addition of ivacaftor 10 μM at time of measurements) to rescue CFTR function.

### Ex vivo MCC assay.

Details can be found in the previous reports (11, 12, 51). Briefly, each whole-length piglet trachea or a CF tracheal trim was cut open along the mid-dorsal line and mounted mucosal side up onto a Sylgard elastomer platform. The prepared trachea was placed into a sealed, highly humidified chamber bubbled continuously with gas (95%/5% O_2_ /CO_2_) with the serosal surface bathed with KRB buffer ± drugs. For the initial 30-minute stabilization period, the tissue was submerged in the bath as the temperature gradually increased to 37°C. Then excess apical solution was drained, and tissue was incubated for an additional 10 minutes before starting baseline measurements of MCCV. Drugs were added by bath replacement with prewarmed bath + drug(s), except for benzamil treatment to inhibit ENaC, which requires apical application (see Supplemental Methods Protocol 1). Summary MCCV data are reported as a single number equal to the average MCCVs measured during the last 50 minutes of the treatment period unless stated otherwise.

### Ex vivo tracheal smooth muscle contraction assay.

Airway smooth muscle contraction was measured as previously described (12). Briefly, piglet tracheal ring preparations of 1.5–2 mm were submerged and securely pinned on a Sylgard-lined Petri dish filled with KRB solution at 37°C and pH 7.4. Digital images of tracheal ring contractions in response to agonists for 1- to 10-minute intervals were recorded with a Nikon digital camera. The cross-sectional surface area of the lumen of the tracheal ring was calculated using ImageJ (NIH).

### X-ray synchrotron assay for ASL depth measurement.

ASL height measurements with ex vivo pig tracheae were done at University of Saskatchewan, Canada, using x-ray synchrotron-based phase contrast imaging (PCI) (29, 30). Briefly, the synchrotron beamline produces sufficient parallel spatial coherence x-rays to increase the contrast between airway lumen and ASL layer by PCI. When x-rays pass through the intact tracheal preparation, the difference in refractive index between ASL and airway lumen results in a phase shift of x-rays that causes a distinctive interference pattern shown on the charge-coupled device detector as variations in x-rays’ intensity. The ASL height measurements were obtained every 5 minutes after the instillation of freshly prepared, custom-built agarose beads (29, 30) which served as a measuring barometer: *t*15-baseline, followed by *t*30-SA (0.3 μM methacholine + 10 μM formoterol). Twenty-four instilled beads from 4 pig tracheae were traced during the baseline and SA treatment periods.

### In vivo sheep MCC assay.

Both in vivo sheep tracheal MCCV and whole lung clearance measurements were done at Mount Sinai Medical Center (MSMC) in a blinded manner. The methods used have been well documented (17). Briefly, to mimic the conditions of inflamed CF airways, sheep were aerosolized with 10 mg of CFTR_inh_172 followed after 2 hours by aerosolization of 2,360 milliunits of *h*NE. This dual treatment typically suppresses tracheal MCCV by 50% from baseline within 4 hours, and it is maintained at least 20 hours. Drugs to be evaluated were aerosolized 4 hours after initial dosing with CFTR_inh_172. MCCV was measured hourly for 8 hours and every 12 hours for up to 24 hours. MCCV following nebulization of methacholine alone was not assessed because of a potential adverse effect.

### Ex vivo MCC and CBF measurements in rat tracheae after in vivo luminal application of agonists.

Rat MCC assay and CBF measurements were done at the University of Alabama using μOCT applied to 6-month-old Sprague-Dawley rats. Measurements were compared for WT and CFTR-*null* rats. After inducing anesthesia by a brief isoflurane inhalation, a rat was administered either a vehicle control or SA (15 μg formoterol + 0.5 μg methacholine) by minimally invasive intratracheal instillation under direct visualization. Rats were euthanized 30 minutes after drug instillation, the chest was opened to explant the trachea, and the trachea was mounted for imaging under μOCT to measure tracheal MCCV and CBF (19).

### pH-stat assay to assess changes of bicarbonate (net base) concentrations in ASL.

The pH-stat method includes an Ussing I_sc_ measurement system and pH electrodes with programmed titrators (Metrohm Titrando 902) (15). All aspects of calibrations of pH electrodes, dosing of titrant (e.g., 5 mM HCl), continuous pH monitoring, and data processing are controlled by Tiamo software (Metrohm). Simultaneous transepithelial I_sc_ is obtained and displayed with a VCC-600 voltage clamp (Physiologic Instruments), and PowerLab Chart4 software (ADInstruments). Tracheal mucosal preparations are mounted in EasyMount Ussing chambers (Physiologic Instruments) with exposed surface areas of 0.5 cm^2^, bathed in Krebs bicarbonate buffer at serosal chambers and bicarbonate-free (replaced by gluconate) unbuffered solution at mucosal chambers at 37°C, and gassed with 95% O_2_ + 5% CO_2_ to serosal and 100% O_2_ to mucosal chambers. Automated dosing device and pH electrode are located at the apical bath. Bicarbonate (net base) secretion rates were measured for unstimulated tissues and in response to SA (10 μM formoterol + 0.3 μM methacholine).

### In vitro multiple particle tracking assay.

The method was described previously (52). Briefly, when the HNEC ALI cultures had reached full maturity, the inserts were cut from their support and placed on a concave well slide filled with media so that only the basal side was exposed to media, and 20 μL of a 0.1% suspension of 2 μm fluorescent polystyrene beads (Thermo Fisher Scientific R0200) was added to the apical surface. The slide was then placed on a custom-built system that includes a heated stage at 37°C and imaged from above with a digital microscope fitted with a high-speed camera (Keyence Inc.). The images were acquired on 3 regions per filter and with tracking of 10–20 beads per region at a frame rate of 1,000 fps. Image files were exported to ImageJ to analyze particle movement with the MTrackJ plugin (v. 1.5.1) of ImageJ. The extracted frame-by-frame coordinates were then used to estimate individual particle distance traveled and MCV. In addition, the coordinates and time lag between frames were used to define time scales (τ) following multiple particle transport methodology (53) and then used to determine the mean squared displacement of the particle for all possible time durations. The D*_eff_* was then calculated from the mean squared displacement and time scales extracted.

### Statistics.

Data are presented as mean ± SEM unless otherwise indicated. We applied statistical analysis (e.g., *P* values) based only on biological replicates, with each individual representing *n* = 1. *P* < 0.05 was considered statistically significant. Results from technical replicates were averaged to obtain the value for each biological replicate. To compare means of different treatment groups, we used 2-tailed paired or unpaired *t* test. For multiple-comparison groups, 1-way ANOVA with Bonferroni’s post hoc test was used.

### Study approval.

The human study was approved by the Institutional Review Board of Stanford University (IRB protocol: 57814), and all individuals provided written informed consent prior to any study procedures. All human nasal tissue and cell culture protocols were approved by the Stanford University IRB (protocol: 42107). All animal tissue protocols in the present study were approved by Stanford IACUC (protocol-10048), by the University of Alabama at Birmingham (protocol-22781), by MSMC (protocol-23-07-A-03), and by the University of Saskatchewan (Animal Use Protocol protocol-20110047) and conducted in accordance with institutional guidelines.

### Data availability.

Values for all data points in graphs are reported in the Supporting Data Values XLS file.

## Author contributions

Conception and design were done by NSJ, JJW, and CEM. Acquisition of data was done by NSJ, XL, JDK, JS, JBB, JRS, MND, and CEM. Analysis and interpretation of data were done by NSJ, SEB, JPI, JS, RWW, JFE, JJW, and CEM. All authors read, reviewed, and approved the final version of the manuscript.

## Conflict of interest

NSJ, JJW, and CEM are listed inventors on a patent pending (19/110,980) covering the use of SA to treat muco-obstructive airway disorders and have founder shares in MCC Therapeutics Inc.

## Funding support

This work is the result of NIH funding, in whole or in part, and is subject to the NIH Public Access Policy. Through acceptance of this federal funding, the NIH has been given a right to make the work publicly available in PubMed Central.

- JFE by NIH (R01 HL165404 and NHLBI Federal Contract 75N92025C00007).
- NSJ, CEM, and SEB by Cystic Fibrosis Foundation (Joo19G0, MILLA23G0, and CFF BIRKET23XX0).
- Stanford Innovative Medicines Accelerator (IMA-1070), Cystic Fibrosis Research Inc., Ross Mosier CF Research Laboratories gift fund, and Galper Family CF fund.

## Supplementary Material

Supplemental data

Supporting data values

## Version 1 04/02/2026

In-Press Preview

## Version 2 06/01/2026

Electronic publication
