# Supplementary material for: Therapeutic potential of synergistic mucociliary clearance for cystic fibrosis airways by β-adrenergic plus cholinergic agonists
Source: J Clin Invest. 2026 Apr 2;136(11):e201541. doi: 10.1172/JCI201541 (PMC13221217; doi:10.1172/JCI201541)
Supplement: Supplemental data [file jci-136-201541-s243.pdf]

SUPPLEMENTARY INFORMATION

Supplementary Figure 1: Sequential synergy agonists prevent airway narrowing.

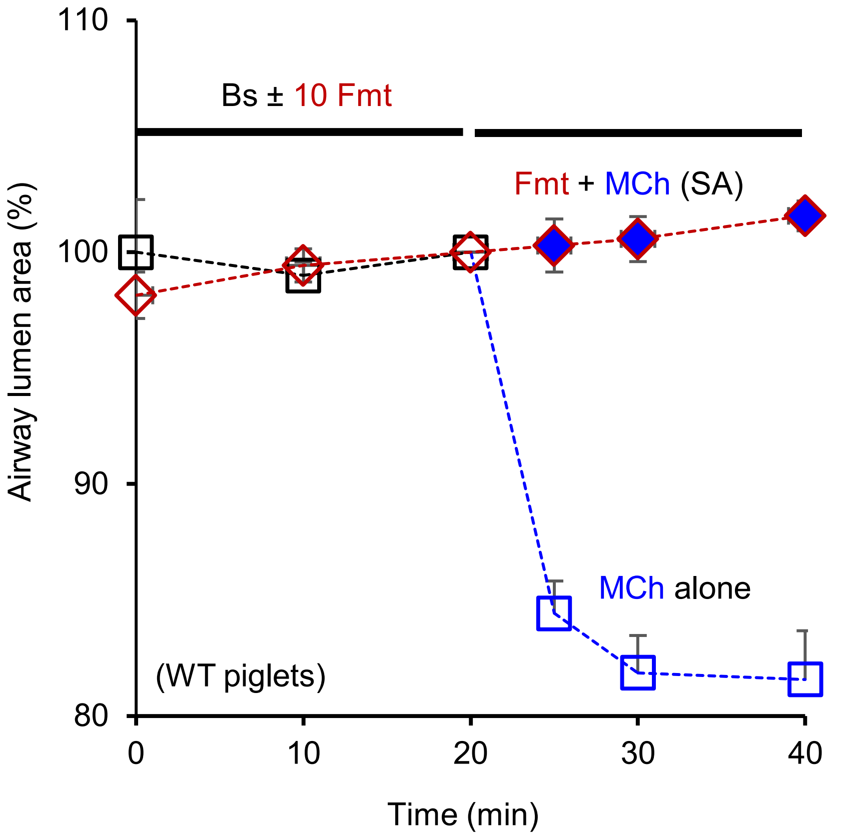

Time courses of ex vivo airway narrowing in response to 0.3 μM methacholine in the presence of 10 μM formoterol pretreatment. Note that methacholine alone induces airway narrowing to 82.6 ± 1.6% from the baseline, while formoterol pretreatment effectively prevent the narrowing during SA period (n = 7 piglets).

Supplementary Figure 2: Atropine fails to induce MCC.

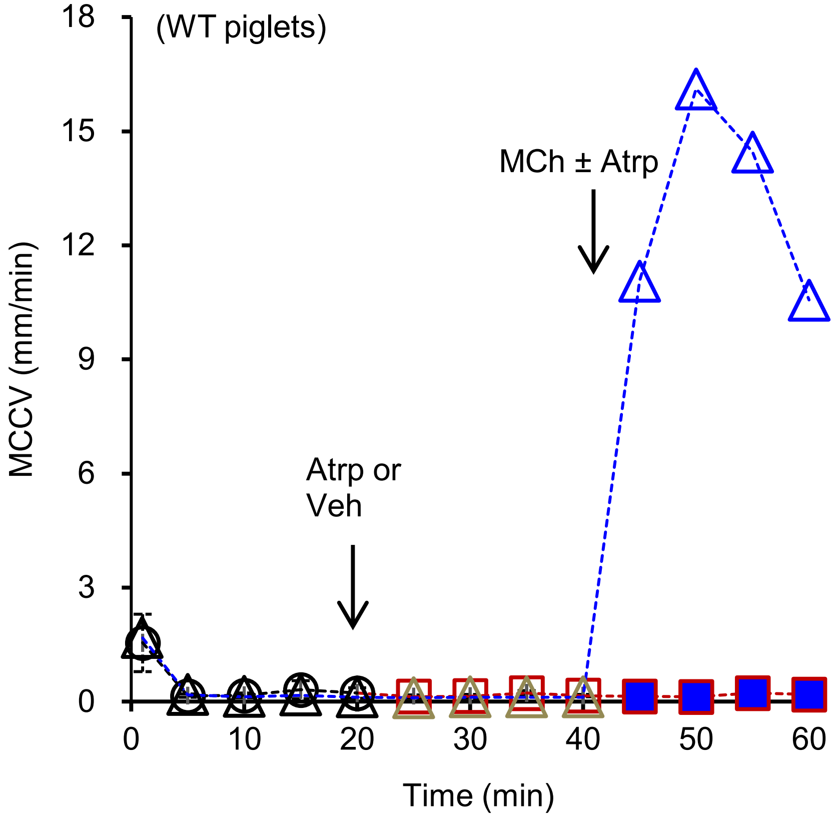

Time course of ex vivo MCCV in the presence of 10 μM atropine (Atrp, red rectangles) or vehicle (0.1% ethanol, tan triangles) shows that atropine abolished 10 μM methacholine (MCh)-induced MCCV (blue triangles) but failed to stimulate MCCV by itself (n = 3 piglets).

Supplementary Figure 3: MCCV by SA is reduced to a half in two hours in ex vivo piglets.

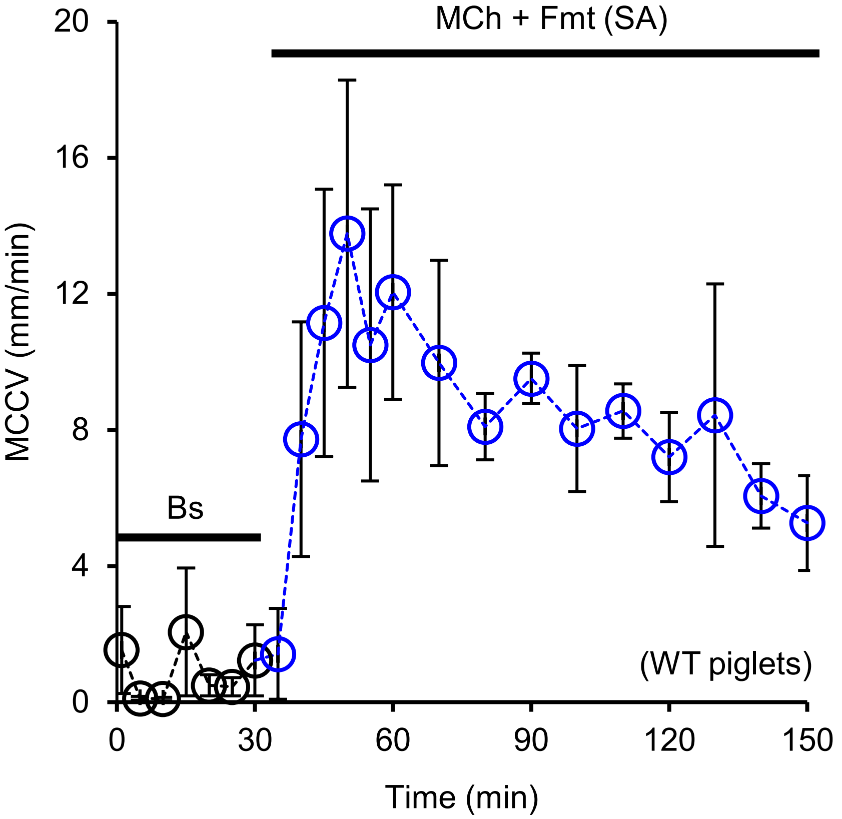

Time courses of ex vivo MCCV shows the synergy agonists-induced MCCV in declined ~50% of its peak value in 2 hours (n = 3 piglets).

Supplementary Figure 4: Synergy agonists improve MCC in small airways.

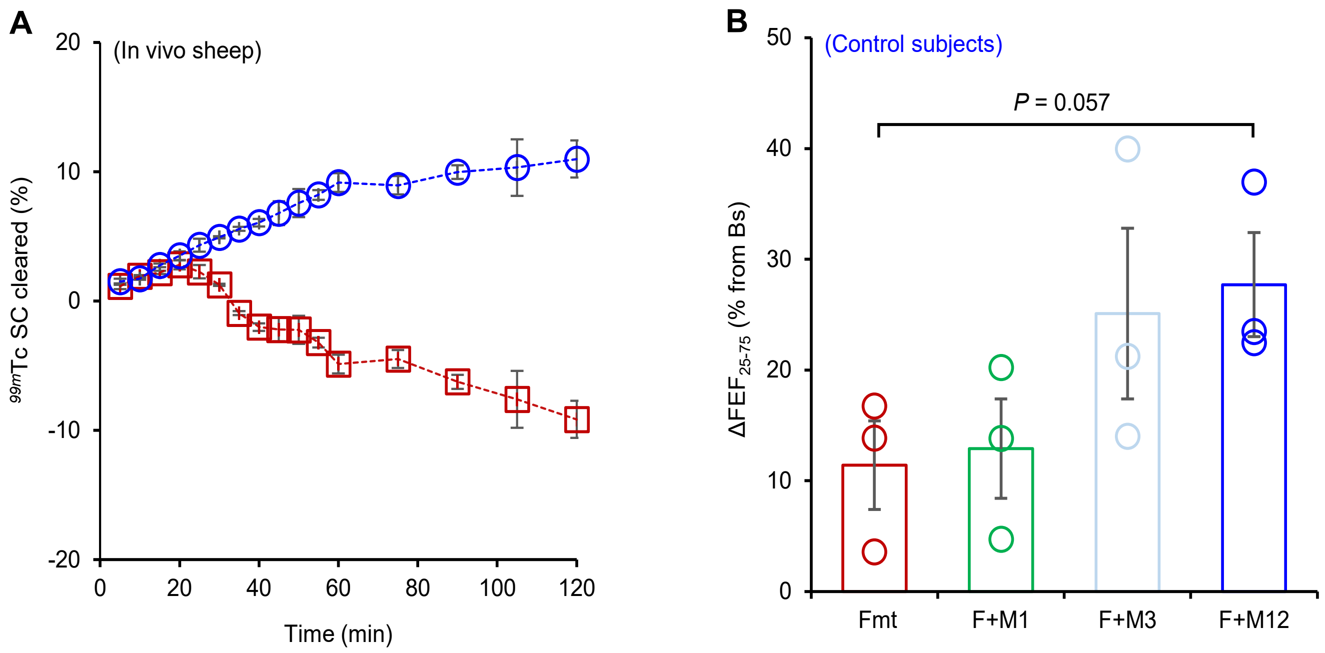

A, Time course of whole lung clearance for 2 hours after SA (20 µg formoterol + 12 µg methacholine) nebulization (blue circles, n = 2 sheep) or vehicle (red squares, n = 2 sheep) following $CFTR_{inh}172$ and human neutrophil elastase (*h*NE), using inhaled radiotracers, technetium labeled sulfur colloid (^{99m}TcSC) and a γ camera. Mean (± SD) clearance of ^{99m}Tc-SC in sheep is expressed as the percentage of the initial deposited lung dose. While vehicle-treated sheep failed to clear the radiotracer, SA induced about 11% clearing of the radiotracer. **B,** In healthy control subjects, SA by nebulization (20 µg formoterol (F) + either 1, 3, or 12 ug methacholine (M)) increases forced expiratory flow at 25–75% (ΔFEF_{25-75}) of the forced vital capacity (FVC), indicating SA improves small airway function (n = 12). A one-way ANOVA with Bonferroni post hoc test was used.

Method for in vivo sheep whole lung clearance

Whole-lung clearance* of a nebulized radiotracer over 2 hours is also measured After $CFTR_{inh}172$ and hNE treatments, sheep are administered aerosol tagged sulfur colloid (^{99m}technetium/TcSC). This is then followed by inhalation of a vehicle (control) or formoterol + methacholine. Measurements via scintigraphy (gamma camera) were taken every 5 minutes for the first hour after and every 15 minutes for the second hour. All deposition images are stored on a computer integrated with the gamma camera. A region of interest is traced over the image corresponding to the right lung of the sheep and the counts recorded. At each time point the counts are corrected for decay and expressed as percentage of radioactivity present in the initial baseline image. The left lung is excluded from analysis because its outlines are superimposed over the stomach and counts can be affected by swallowed radiolabeled mucus. Note that only a couple of sheep was assessed by whole lung clearance assay because of prohibitive costs.

*Sabater, J. R. *et al.* Aerosolization of P2Y(2)-receptor agonists enhances mucociliary clearance in sheep. *J Appl Physiol (1985)* **87**, 2191-2196, doi:10.1152/jappl.1999.87.6.2191 (1999).

Supplementary Table 1. Baseline demographic of study participants for single dose tolerability trial of synergy agonists.

	**Overall**	**Non-CF (Control)**	**CF**
**N**	38	13	25
**Age (years, mean/SD)**	31.3 ± 1.4	37.4 ± 4.4	28.1 ± 8.0
**Sex**	17 M : 21 F	3 M : 10 F	14 M : 11 F
**Race**	33 W 5 A	9 W 4 A	24 W 1 A
**Ethnicity**	4 H 34 NH	1 H 12 NH	3 H 22 NH
**CFTR genotype (n, %)**<br><br>Hom. F508del<br>Het. F508del<br>Het. Non-F508del		 <br>N/A	 <br>12 (48%)<br>7 (28%)<br>6 (24%)
**History of airway reactivity**	9	0	9 (37%)
**On HEMT**		N/A	25 (100%)

M, male; F, female; W, white; A, Asian; H, Hispanic; NH, non-Hispanic; HEMT, highly effective modulator therapy

Supplementary Protocol 1

Protocol for ENaC inhibition ex vivo piglet trachea MCC assay.

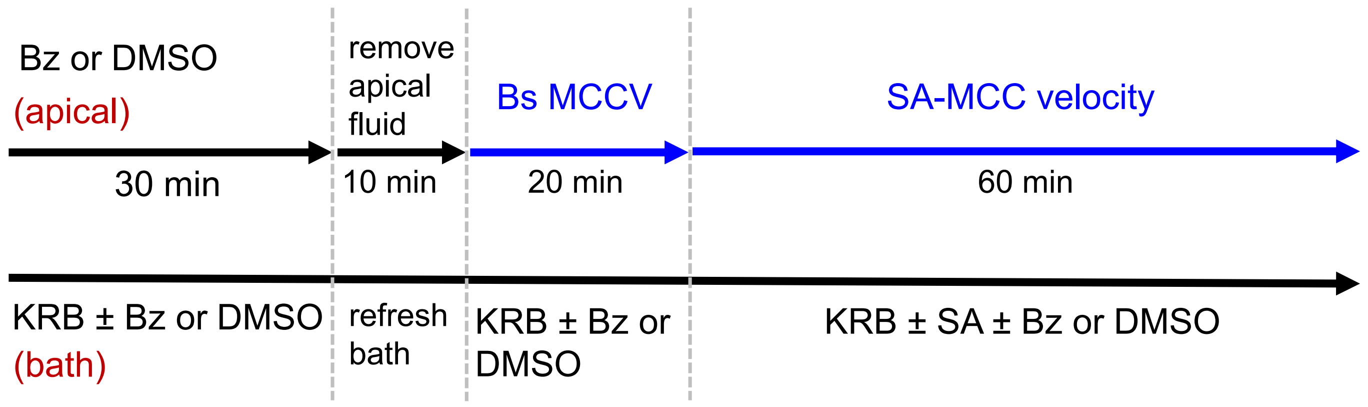

Bz, 10 μM benzamil; Bs MCCV, baseline MCCV; SA, synergy agonists, 10 μM formoterol + 0.3 μM methacholine.
